# Effect of dorsal nerve fascial island flap on repairing distal soft tissue defects at the proximal segment of the index, middle, ring, and little fingers

**DOI:** 10.1186/s13018-022-03309-9

**Published:** 2022-09-14

**Authors:** Xun Wang, Jingdan Lv, Shujiao Liu, Shuangyue Xu, Guoliang Yan

**Affiliations:** 1 Hand and Foot Surgery Department, The Third Hospital of Xiamen, The First Affiliated Hospital of Xiamen University (Tongan Branch), Xiamen City, 361100 Fujian Province China; 2 Department of Nosocomial Infection Control, The Third Hospital of Xiamen, The First Affiliated Hospital of Xiamen University (Tongan Branch), Xiamen City, 361100 Fujian Province China; 3 School of Medicine, Xiamen University, Xiamen City, 361005 Fujian Province China

**Keywords:** Dorsal nerve fascial island flap, Soft tissue defect, Dorsal branch of proper palmar digital artery, Dorsal digital nerves, Hand surgery

## Abstract

**Background:**

To investigate the effect of the dorsal nerve fascial island (DNFI) flap on repairing finger soft tissue defects at the distal segments.

**Methods:**

Fifty patients with distal soft tissue defects at the index, middle, ring, or little fingers were treated with a DNFI flap at the proximal phalanx between February 2008 and May 2018. The nutrient vascular chain around the dorsal branch of the proper palmar digital nerves served as the flap axis. The dorsal branch of the proper palmar digital arteries provided blood supply. The fascia pedicle served as the venous system. All patients were followed for 6 months.

**Results:**

All 50 flaps survived. The appearance, color, and texture of the skin returned to normal. The sensory function was partially restored. The two-point discrimination of the finger flap was 7–10 mm.

**Conclusions:**

The DNFI flap at the proximal phalanges of the index, middle, ring, and little fingers is an effective surgical option. The technique has a high flap survival rate and long pedicle, which can repair different parts of the finger. The flap also restores the sensory function of the finger without damaging the main nerves or blood vessels. The flap treatment is an optimal option for finger soft tissue defects at the distal segments.

## Introduction

Injury at the distal segment of the finger is a common hand injury often accompanied by tendon and phalanx exposure. The major treatment method is to cover the wound with a skin flap of similar texture, without changing the length of the phalanx. This strategy aims to restore the sensory function and normal appearance of the finger, as well as minimize damage to the main nerves and blood vessels at the donor site. Different types of flaps have been designed based on this method; however, each has some drawbacks. The cross-finger flap or the thenar flap restricts the finger joint for an extended period of time, leading to joint stiffness and a second surgery [1]. The abdominal embedding flap similarly restricts the finger, resulting in stiffness and swelling, and also requires a second surgery [2]. The disadvantages of a free flap include long and complicated procedures, as well as a high failure rate [3, 4]. The reversed digital artery flap contains an abscised proper palmar digital artery (PPDA) and therefore may significantly damage the artery [5, 6]. In this study, we reviewed 50 patients who had distal soft tissue defects at the index, middle, ring, or little finger and treated them with a dorsal nerve fascial island (DNFI) flap at the proximal phalanx. Our results showed that this treatment method is safe and simple, requires shorter surgical time, and has a high flap survival rate. Owing to the high flexibility, this flap may be used for repairing soft tissue defects in different parts of all fingers.

## Methods

### General patient information

**Fig. 1:**
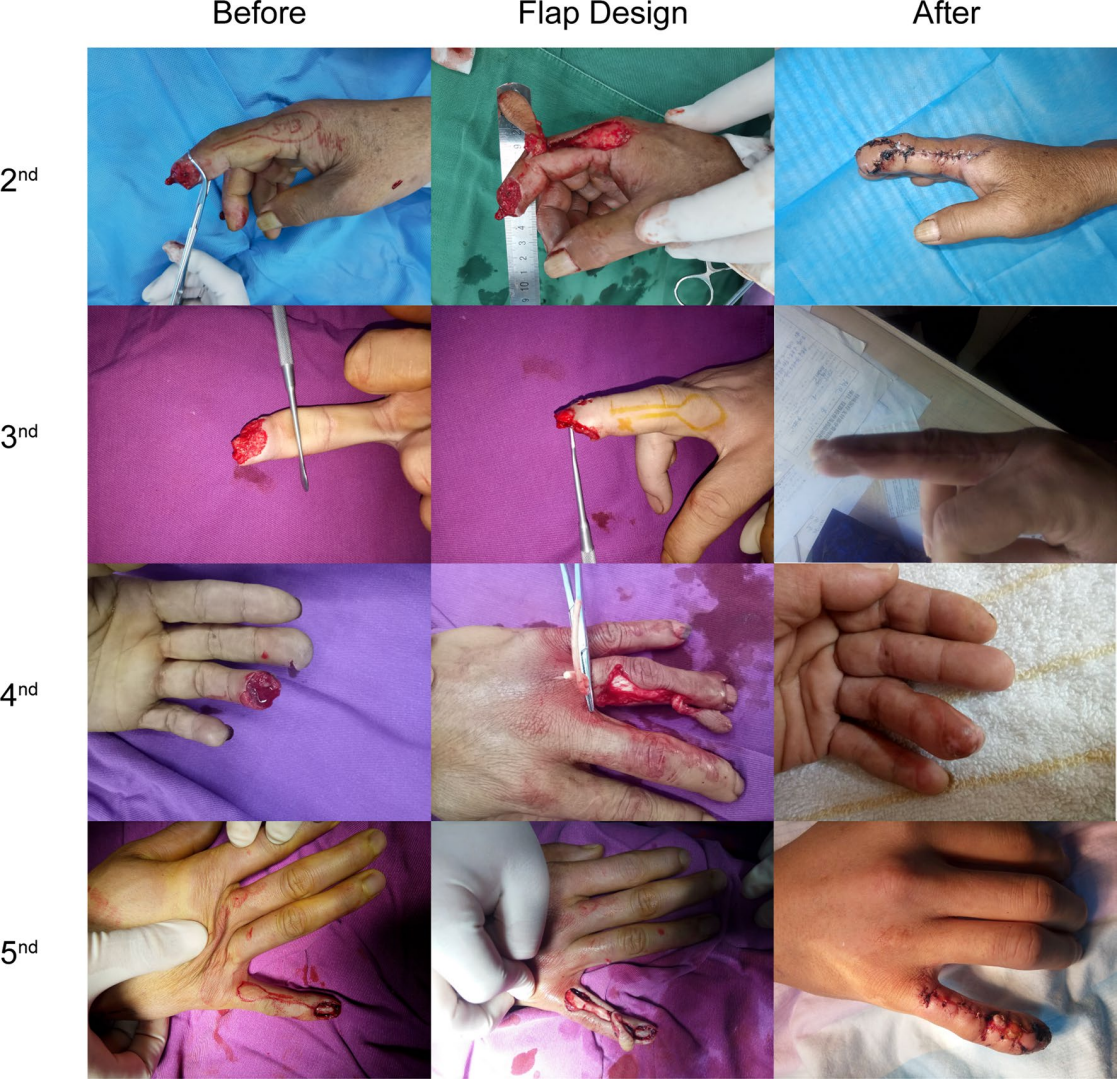
General information of each finger surgery

A total of 50 patients (41 males and 9 females) with an average age of 35 years (range 18–65 years) were included. There were 30 cases with crushing injury, 11 cases with cut injury, and 9 cases with wringing injury. Fifteen patients had index finger injury, 14 had middle finger injury, 12 had ring finger injury, and 9 had little finger injury. The defect size was between 1.0 cm × 1.0 cm and 2.0 cm × 2.5 cm with bone or tendon exposure (Fig. 1).

### Surgical methods

The DNFI flap is suitable for the recipient site of the middle and distal phalangeal segment of the index, middle, ring, or little finger with a defect size of less than 2.5 cm × 2.0 cm.

### Flap design

**Fig. 2:**
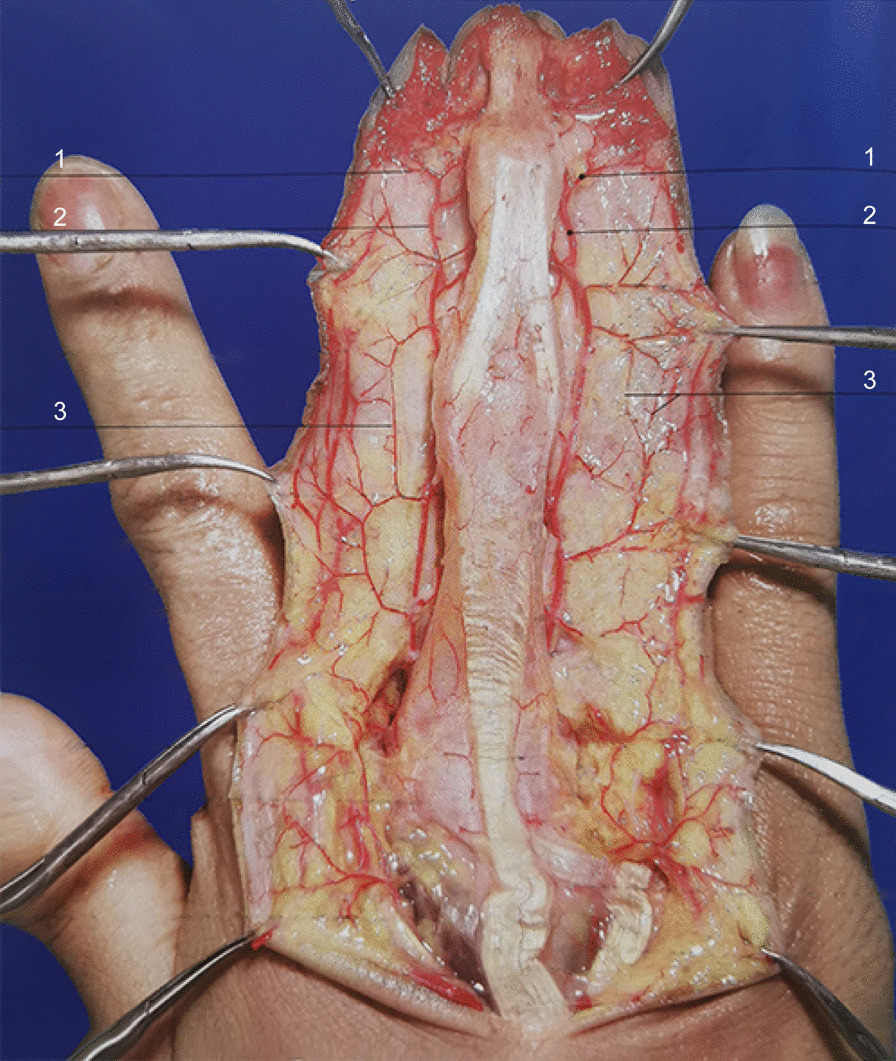
Dorsal branches of digital arteries. 1: Dorsal branches of digital arteries; 2: proper palmar digital arteries; 3: cutaneous branches of dorsal branches

**Fig. 3:**
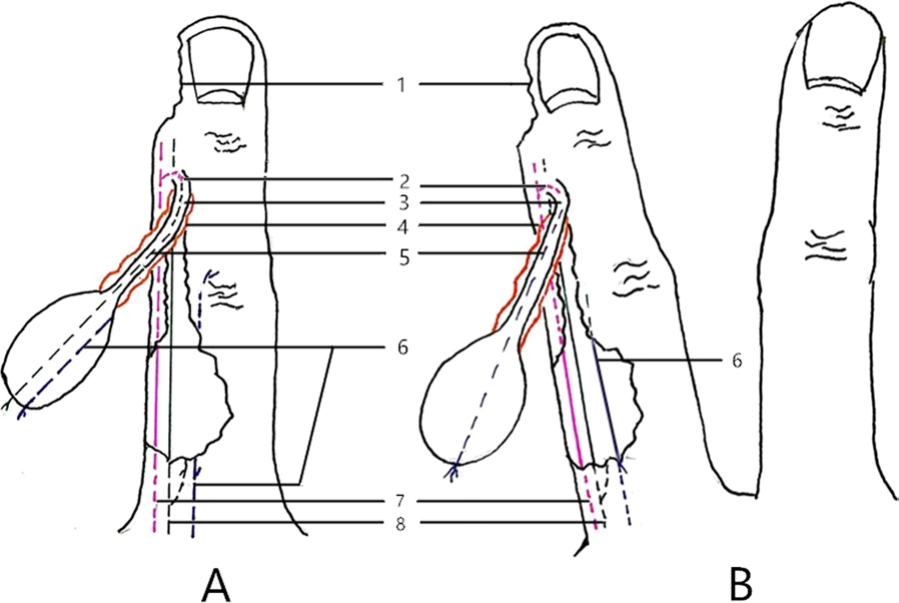
The schematic of flap extraction. **A**: Middle finger; **B**: index, ring, and little finger. 1. Defect region; 2. dorsal branch of the PPDA; 3. skin strip; 4. subcutaneous fascia; 5. dorsal branch of the PPDN; 6. dorsal digital nerve developed from the metacarpal dorsal nerve; 7. PPDA; 8. PPDN

The flap was designed according to the size, shape, and position of the defect using the point-line-plane method [7]. *Point*: The distal end of the middle phalanx served as the rotation point, also known as the outlet point of the arterial branch (the distal inter-phalanx joint branch), which originates from the PPDA towards the dorsal side at the phalanx neck depression of the distal end of the middle phalanx [8] (Fig. 2). *Line*: The dorsal edge of the ulnar or radial side served as the axis. *Plane*: The area between the dorsal midline of the proximal phalange and the radial or ulnar edge of the palmar side of the finger served as the plane. According to the point-line-plane method, the range of the flap was between the metacarpophalangeal and the interphalangeal joint, while the pedicle was at the distal end of the middle phalanx (Fig. 3).

### Flap cutting

Patients were administered brachial plexus anesthesia. A tourniquet was applied to the upper arm to occlude blood flow. The skin and subcutaneous tissues above the tendon were incised. The aponeurosis of the tendon was protected in case of subsequent skin grafting. The flap, which was above the aponeurosis, was cut proximally, making sure that the skin was not separated from subcutaneous tissues. The dorsal nerve at the proximal segment of the flap was selected based on the dorsal nerve distribution of each finger and then cut. It included the dorsal digital nerves originating from the superficial branch of the radial nerve (SBRN) or the dorsal branch of the ulnar nerve (DBUN) and the dorsal branches of the proper palmar digital nerve (PPDN) in most cases. The entire dorsal nerve surrounded by the nutrient vascular chain was included in the flap and the fascial pedicle to achieve reliable blood supply and circulation, as well as a high flap survival rate. The flap was then cut proximally to distally according to the travelling line of the selected dorsal nerve and contained the entire nerve.

### Management of fascial pedicle

The pedicle was located at the dorsa-lateral portion of the middle phalanx. To prevent venous flow obstruction, fascial tissues in the middle segment were at least 6–8 mm in width and with a depth above the extensor tendon. The blood supply of the flap was mainly from the dorsal branches of the PPDA. To balance the blood flow between arteries and veins, the fascial and soft tissues around the rotation point, also known as the fascial pedicle root at the dorsal branch of the PPDA, were spared as much as possible and closer to the palmar side.

### Skin flap transfer

The area of the flap was at least 15% larger than the wound surface to reduce the tension after suture. To avoid blockage of blood vessels, the skin between the rotation point and the wound surface was incised, and the edges of the skin on both sides of the incision were dissociated until the space below was sufficient for the rotated fascial pedicle. The skin on the surface of the fascial pedicle of the middle phalanx was then spared at 3–4 mm. After the flap was turned over to cover the wound, the skin strip of the pedicle was sutured with the skin edges on both sides, making sure that the pedicle was not compressed. The wound was then covered with the flap and sutured. If the cutaneous nerve in the defect could be found, the cutaneous nerve in the defect area was anastomosed with the nerve in the flap to restore the sensory function of the finger. The proximal donor site was sutured or covered with the skin grafts according to the size of the donor site.

### Postoperative management

Patients were instructed to stop smoking for one month after surgery and to minimize movement of the repaired finger as much as possible. In winter, the fingers were kept warm by an infrared paint curing lamp. Patients were also instructed to raise the affected limb higher than the heart to promote venous return for about 2 weeks until the flap survived. The pedicle should remain loose. The suture was removed when severe swelling occurred. All patients were given anti-inflammatory, anti-thrombotic, and anti-angiogenic treatments for 5 days.

### Measurement of sensory function recovery

The static 2-PD test was performed to quantitatively evaluate sensory function recovery using a caliper with two small needles. Patients were instructed to close their eyes and touch the needles on the caliper against the skin at different distances from each other. The test determines the minimal distance at which the patient cannot distinguish two points in contact with the skin. The normal distance is 3–5 mm. The closer the defected finger to the normal range, the better the recovery of the sensory function.

## Results

All 50 flaps survived. Five cases presented with blisters in the middle and distal segment of the flap after surgery. After 8–10 days of preventing the blisters from rupturing, the blisters were gradually absorbed. One patient presented with epidermal necrosis, but the deep layer survived, and the wound healed after 4 weeks of dressing changes.

All patients were followed for an average of 8 months (6–18 months). We performed the static 2-PD test on 20 patients, who were then divided into two groups: one group with 5 patients who received nerve anastomosis and the other group with 15 patients who did not. The mean 2-PD values of the two groups were 7.8 mm and 7.9 mm, respectively, suggesting that neurorrhaphy did not markedly promote sensory recovery in patients with the DNFI flap. Therefore, the 2-PD test was not performed on the subsequent 30 patients. Although the range of 2-PD value was 7–10 mm, which was shorter than the normal range (3–5 mm), it was negligible in daily life according to the patients. All 50 patients were satisfied and used the digits without difficulty. The appearance, color, and texture of the skin recovered. The swelling disappeared, and there were no obvious complications. No patient needed secondary treatment for flap reconstruction or thinning.

### Case report

**Fig. 4:**
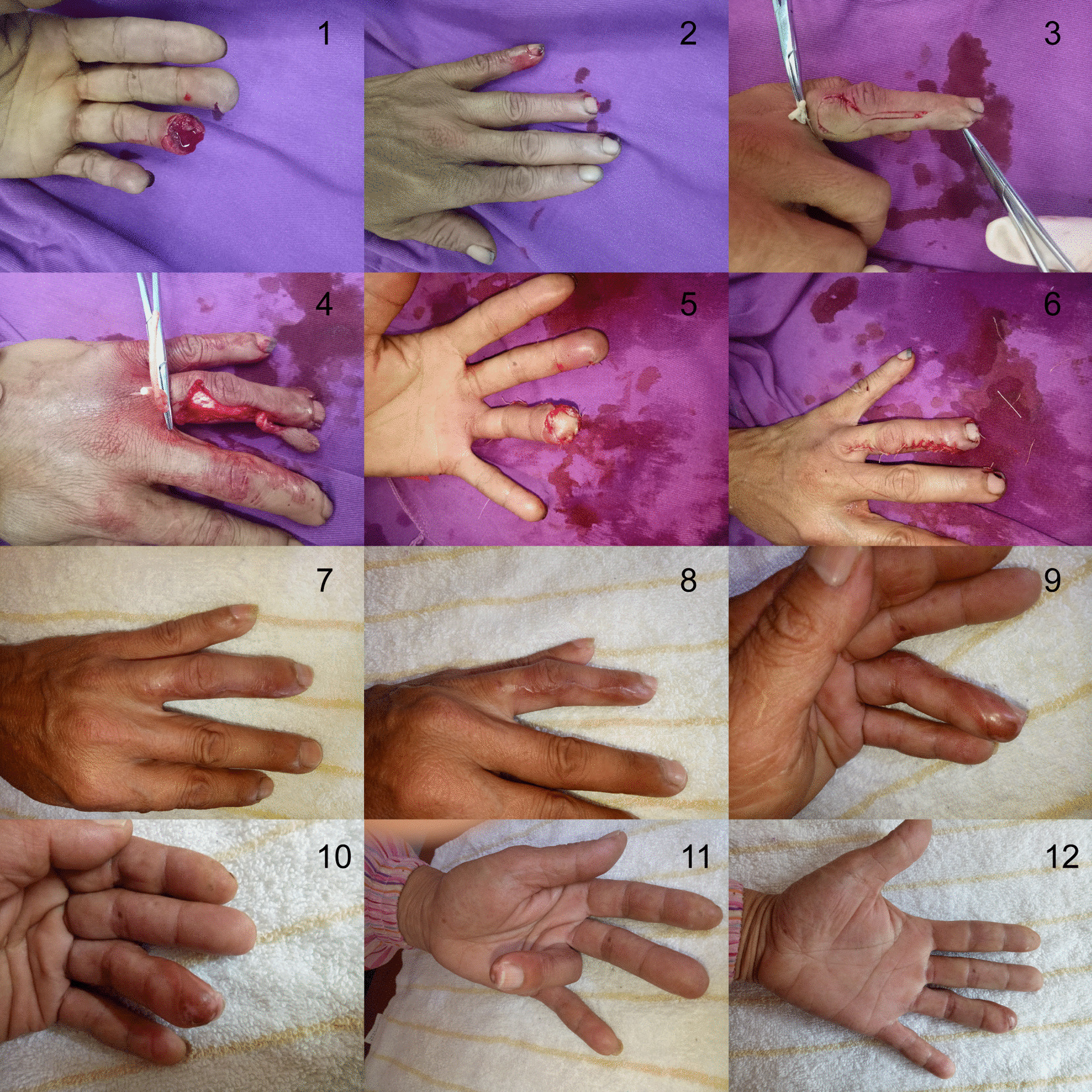
Case presentation. 1. Pre-operative ventral side; 2. pre-operative back side; 3. flap design; 4. flap dissection; 5. wound repair; 6. donor site suture; 7–12. Six months after surgery; 7: postoperative back side; 8. postoperative donor site; 9. postoperative redial side; 10. postoperative ventral side; 11. recovery of finger flexion function; 12: recovery of finger stretch function

The patient was a 58-year-old female. The palmar side of the terminal phalanx of the left ring finger was mechanically crushed, and part of the phalanx bone was exposed for 2 h. The size of the defect was 1.5 cm × 1.5 cm on the palmar side. The DNFI flap at the radial side of the ring finger was designed (1.8 cm × 2.0 cm), with the rotation point on the distal end of the middle phalanx, which was 0.5 cm from the terminal-middle phalanx joint. The flap contained the dorsal digital nerve stemming from the DBUN. After the transfer, the dorsal digital nerve was anastomosed with the sensory nerve in the defect area to facilitate flap recovery. The donor site of the flap was sutured directly. After surgery, the flap survived (Fig. 4). At 6-month follow-up, the appearance and texture of the flap recovered, and the static 2-PD value was about 7 mm.

## Discussion

**Fig. 5:**
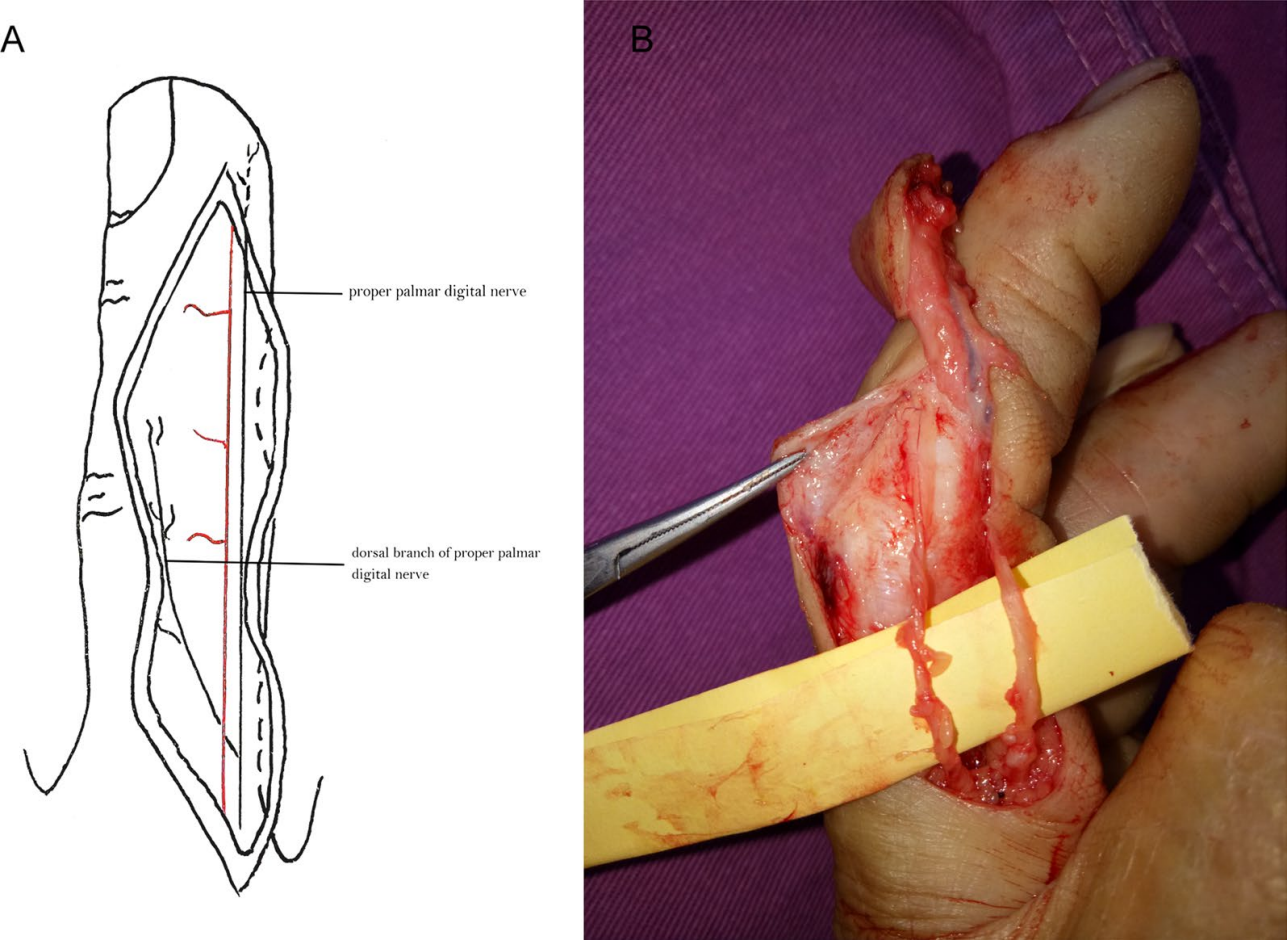
The dorsal nerves include the dorsal branches of the proper palmar digital nerve. **A**. Schematic presentation; **B**. surgery image

**Fig. 6:**
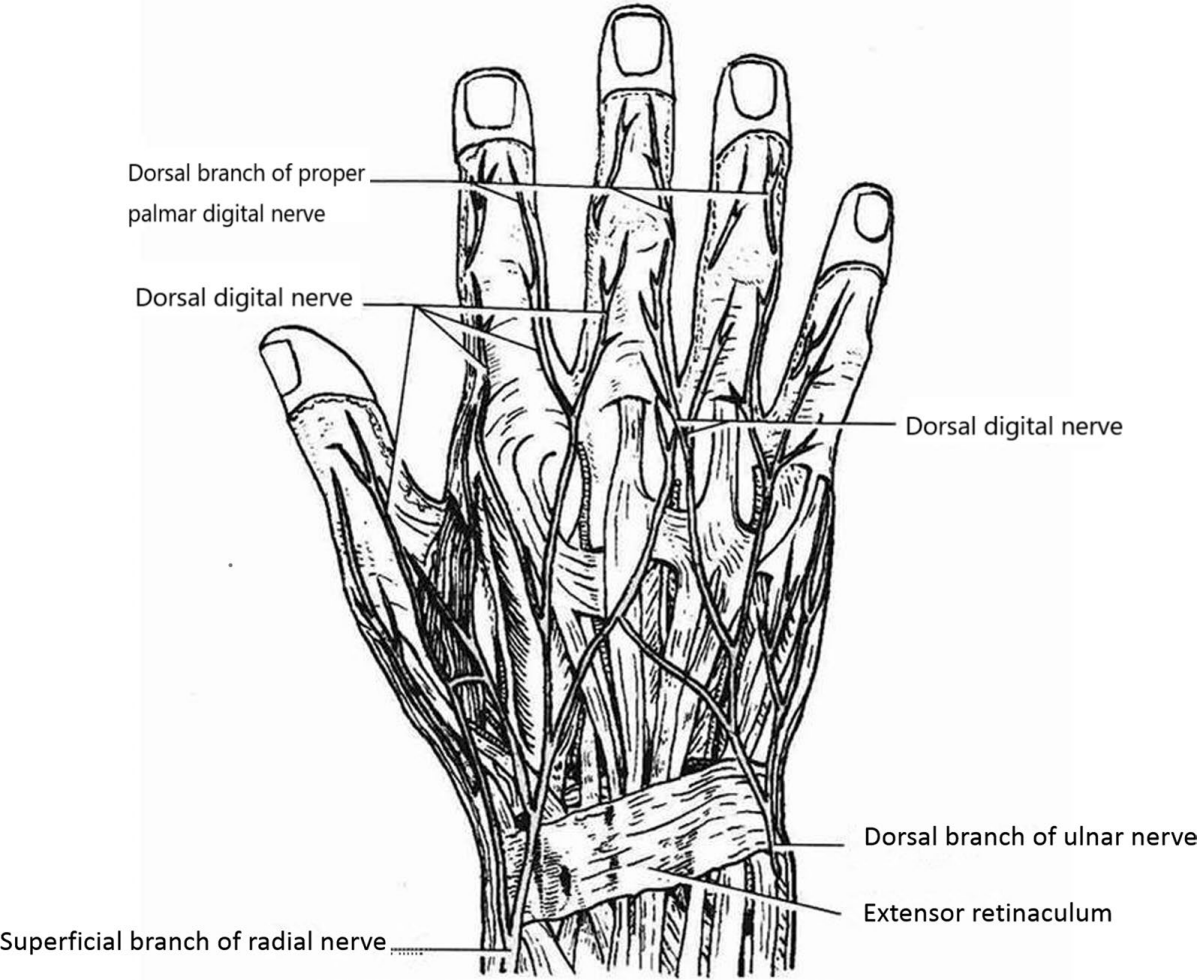
Schematic of the dorsal branches of the radial and ulnar nerve

The dorsal nerves of the index, middle, ring, and little fingers include the dorsal branches of the PPDNs (Fig. 5) and the dorsal nerves originated from the SBRN and the DBUN (Fig. 6) [9–16]. At the midportion of the proximal phalanges [13, 15] or the metacarpophalangeal joints [16] of the index, middle, and ring fingers on the palmar side, the PPDNs constantly develop large dorsal branches on both the ulnar and radial sides of each finger, which run towards the dorsum of the finger [12, 15]. Two branches of each finger gradually run closer to each other towards the back midline, beginning at the middle segment of the proximal phalanx and ending at the dorsal side of the final interphalangeal joint or distal phalanx [9]. The PPDN of the little finger seldom develops dorsal branches on both sides [9]. The SBRN develop dorsal digital branches towards the dorsal skin of the index finger and towards the dorsal radial half skin of the middle finger, all of which end at the proximal phalanges [10, 11]. The DBUN develops dorsal digital branches towards the dorsal ulnar half skin of the middle finger, and the dorsal skin of the ring finger, all of which end at the proximal phalanges [11]. The DBUN also develops dorsal digital branches towards the dorsal skin of the little finger, which end at the distal phalanx [9]. The flaps of the index, middle, and ring fingers must include the dorsal digital branches of SBRN or DBUN and the dorsal branches of the PPDN to maintain continuity of the nervous system, thereby improving the survival of the flap. The PPDN of the little finger seldom develops dorsal branches. Also, the dorsal digital branches of the ulnar nerve run all the way to the dorsum of the little finger. Accordingly, the flap of the little finger only includes the dorsal digital branches from the ulnar nerve. Compared with the complex innervation of the index, middle, ring, and little fingers, the origin and distribution of the dorsal digital nerves of the thumb are constant and the diameter of the nerves is larger. A recent retrospective cohort study showed that coaptation of the dorsal digital nerves originated from SBRN with the PPDN in the thumb pulp defect was superior to non-coaptation in sensory recovery [17]. The discrepancy between the results of the above study and ours may be due to the differences in the dorsal nerves of the thumb and other fingers.

The use of the dorsal flap at the index finger for repairing a wound on the thumb was first proposed by Foucher et al. [18]. Subsequently, a number of anatomical studies on the dorsal branches of the PPDAs have been performed [8, 19–22]. It has been found that the PPDAs at the index, middle, ring, and little fingers develop two relatively constant large branches at the proximal and the middle phalanges, respectively [8, 19, 21, 22]. One of the middle phalanx branches is at the middle segment, and the other is at the distal segment towards the distal interphalangeal joint [8, 21, 22] (Fig. 2). The branch at the middle of the middle phalanx or those at the distal interphalangeal joint were included in the flap to provide blood supply to the wound. The ascending and descending branches originating from the dorsal branches of the PPDAs communicate with adjacent dorsal digital arteries, develop smaller vessels, and thus form an arterial network in the subcutaneous fascia layer along the dorsal nerves [7, 8]. The arterial network allows the nerve-included flap to be cut much longer than other methods. In addition, the tiny venules and the venous network accompanied by the arterial network seemed to be sufficient for venous drainage [8, 12].

The DNFI flap at the proximal segment of the index, middle, ring, and little fingers is a simple, convenient surgical option with a high success rate. It is superior to other flaps for repairing distal soft tissue defects. The cross-finger flap damages the neighboring donor finger. The cross-finger flap and the thenar flap restrict the injured finger joint for 3–4 weeks, leading to joint stiffness and a second surgery [1, 23]. In contrast, the donor site and the recipient site of the DNFI flap are on the same finger. Therefore, the neighboring fingers will not be fixed or damaged, which reduces pain. The texture and color of the donor skin of the cross-finger flap and the DNFI flap were similar to the affected skin, which appeared to be in good shape with high rub fastness after surgery. Both flaps are superior to the thenar flap in terms of the flap shape and rub fastness. The abdominal embedding flap also restricts the finger joints and the affected upper limb joints, resulting in joint stiffness and swelling, and requires a second surgery [2]. Since the skin texture of the abdomen is quite different from that of the finger, the abdominal flap shows lower rub fastness than the DNFI flap. However, in cases with larger defects or multi-defects on fingers and hands, the abdominal flap may be a better choice [24, 25]. Another fingertip reconstruction method is the free flap. The disadvantages of this method include long and complicated procedures, as well as a high failure rate. It requires surgeons with abundant microsurgery experience [3, 4]. Compared with the free flap, the DNFI flap depends less on the microsurgery experience of surgeons and has a higher success rate, a lower surgery risk, and a shorter operation period. In cases with small defects on the distal segment of the fingers, the DNFI flap may be more convenient and effective. The digital artery flap is also a common method to repair finger defects. The reversed digital artery flap contains an abscised PPDA and therefore may significantly damage the artery [5, 6]. The DNFI flap harvests the dorsal branch of PPDA and PPDN, without sacrificing the main trunk of the nerves and arteries of the hand, which reduces the damage to the natural blood supply. As there is a complex vascular network in the subcutaneous tissues of the finger [8, 12, 21], many fascia flaps have been developed and widely used [8, 26, 27]. These flaps, including the DNFI flap, contain the dorsal branch of the PPDA and the arterial network as the blood supply, as well as tiny venules and the venous network which serve as the venous drainage system. The pedicle of the DNFI flap is located at the dorsa-lateral portion of the middle phalanx, while that of the traditional fascia flap is at the dorsum of the middle phalanx. This design reduces the damage to the dorsal extensor tendon and avoids scars on the dorsal skin of the proximal interphalangeal joint, thus optimizing joint mobility. In addition, the flap is at the proximal phalange. The skin and the subcutaneous tissues are loose here; therefore, the small proximal donor site can be sutured without skin grafting after flap-cutting. The DNFI flap is also long enough to reach the soft tissues and the skin defects on any side of the finger due to the location of the pedicle and the flap. Lastly, the skin on the fascial pedicle was spared at 3–4 mm. After the flap was transferred, the skin strip of the pedicle was sutured with the skin edges on both sides. A loose and wide pedicle channel was then formed, which was adequate to accommodate more fascia tissues with more small vessels, strengthening the blood supply and venous drainage of the flap [8, 12]. A loose channel also reduces pedicle tension after the suture, which improves flap survival. Additionally, the nutrient vascular network around the dorsal nerves enhances arterial blood perfusion [7].

The disadvantages of the DNFI flap method should also be noted. First, the flap requires a wide fascial pedicle; therefore, the flap may be swollen after surgery. However, tumefaction usually disappears, and the skin recovers 6–8 weeks after surgery. Secondly, the flaps may occasionally induce blisters due to venous flow obstruction. In our patients, blisters were gradually absorbed within 8–10 days after surgery. Even if surface necrosis occurs, the sub-surface layer can survive by regular dressing changes. Thirdly, the sensory function of the finger may not be completely restored even though an anastomosis between the nerves in the flap and the wound is achieved.

Surgeons should be familiar with the distribution of the dorsal nerves, and the dorsal branches of the PPDA to ensure that the branches of the blood vessels will not be damaged. The fascial pedicle should also be spared as much as possible. To keep the pedicle loose and uncompressed, the skin on the surface of the fascial pedicle of the middle phalanx was spared at 3–4 mm. The incision between the rotating point and the wound surface must be cut straight, and the edges of the incision should be disassociated to accommodate the reversed pedicle. Thirdly, the suture of the pedicle and the flap to the skin edges should be loose, and the distance between each stitch should be wide to avoid excessive tension. Lastly, if blisters appear due to venous flow obstruction, part of the suture of the pedicle or the flap to the skin edges can be removed. Small blisters can be absorbed without treatment, while large blisters need to be drained by a syringe. The outer membrane of the blisters should be protected from damage and loss.

## Conclusions

In conclusion, the DNFI flap is a promising surgical option with a high flap survival rate, little damage, and a wide repair range. The repaired fingers showed good appearance, color, texture, and rub fastness. Our study indicates that the DNFI flap is a feasible surgical option for finger soft tissue defects at the distal segment.

## Data Availability

The datasets generated and analyzed during the current study are not publicly available due to limitations of ethical approval involving the patient data and anonymity but are available from the corresponding author on reasonable request.

## Abbreviations

DBUN: Dorsal branch of the ulnar nerve
DNFI: Dorsal nerve fascia island
PPDA: Proper palmar digital artery
PPDN: Proper palmar digital nerve
2-PD: Two-point discrimination
SBRN: Superficial branch of the radial nerve
